# Reconstructing functional brain networks: have we got the basics right?

**DOI:** 10.3389/fnhum.2014.00107

**Published:** 2014-02-27

**Authors:** David Papo, Massimiliano Zanin, Javier M. Buldú

**Affiliations:** ^1^Computational Systems Biology Group, Center for Biomedical Technology, Universidad Politécnica de MadridMadrid, Spain; ^2^Departamento de Engenharia Electrotecnica, Faculdade de Ciencias e Tecnologia, Universidade Nova de LisboaLisboa, Portugal; ^3^Innaxis Foundation & Research InstituteMadrid, Spain; ^4^Laboratory of Biological Networks, Center for Biomedical Technology, Universidad Politécnica de MadridMadrid, Spain; ^5^Departamento de Tecnología Electrónica, Universidad Rey Juan CarlosMóstoles, Spain

**Keywords:** complex networks theory, functional brain networks, correlations, synchronization, data mining

Both at rest and during the executions of cognitive tasks, the brain continuously creates and reshapes complex patterns of correlated dynamics. Thus, brain functional activity is naturally described in terms of networks, i.e., sets of nodes, representing distinct subsystems, and links connecting node pairs, representing relationships between them.

Recently, brain function has started being investigated using a statistical physics understanding of graph theory, an old branch of pure mathematics (Newman, 2010). Within this framework, network properties are independent of the identity of their nodes, as they emerge in a non-trivial way from their interactions. Observed topologies are instances of a network ensemble, falling into one of few universality classes and are therefore inherently statistical in nature.

Functional network reconstruction comprises various steps: first, nodes are identified; then, links are established according to a certain metric. This gives rise to a clique with an all-to-all connectivity. Deciding which links are significant is done by choosing which values of these metrics should be taken into account. Finally, network properties are computed and used to characterize the network.

Each of these steps contains an element of arbitrariness, as graph theory allows characterizing systems once a network is reconstructed, but is neutral as to what should be treated as a system and to how to isolate its constituent parts.

Here we discuss some aspects related to the way nodes, links and networks in general are defined in system-level studies using noninvasive techniques, which may be critical when interpreting the results of functional brain network analyses.

## What's a node?

A node is a drastically coarse-grained representation of an object, identifying it to a structureless point, in a way similar to the reduction of a whole mechanical system to its center of mass, allowed by the system's symmetries.

Identifying nodes supposes that the studied system can meaningfully be decomposed into different parts, a challenging task when dealing with spatially extended systems of largely unknown organization and complex dynamics.

Defining a node generates qualitatively different problems for different recording techniques: for non-invasive system-level electrophysiological techniques, the main issue is how well sensors sample the underlying dynamical system; for functional magnetic resonance imaging ones, the central question is how to best segment the space.

### Sub-sampling

Studies using electrophysiological techniques such as electro- (EEG) or magnetoencephalography (MEG) identify nodes with sensors and, as a consequence, drastically undersample electrical activity at a neuronal level and the corresponding functional space.

The spatial sampling implicitly leads to a coarse graining of the dynamics, introducing a spatial scale irrespective of the actual system organization, resulting in spatial correlations in the topology of reconstructed networks.

Even more importantly, sub-sampling can severely affect topological network properties (Stumpf et al., 2005; Lee et al., 2006). While the functional networks based on synchronization of MEG sensors may be qualitatively similar to those obtained after source reconstruction in the anatomical space (Palva et al., 2010), network topologies derived from surface recordings may not reflect the topology of the underlying network of neuronal sources (Antiqueira et al., 2010), let aside that of anatomical connections between them (Ponten et al., 2010).

Limitations in the amount of data and in the reliability of link estimation (either due to the presence of noise, of common sources or the inability of most estimators to distinguish between direct and indirect interactions with the same dynamical subsystem) likely lead to the spurious addition, deletion or changes in the nature of links. Spurious links between nodes of similar degree may for instance decrease the average shortest path length and increase the clustering coefficient (Lee et al., 2006). As a result, networks may erroneously be classified as small-world and assortative, even when their true structure is disassortative (Bialonski, 2012).

Furthermore, randomly sub-sampled scale-free networks generally turn out not to be scale-free (Stumpf et al., 2005), and multiple electrode recordings generally overestimate the true network small-worldness, as each sensor picks up many sources at small scales, while their number constrains the sampling on large ones (Gerhard et al., 2011).

### Parcellation

For functional magnetic imaging data, delineating functionally separated brain units, a task that goes under the name of *parcellation*, may be nontrivial (Stanley et al., 2013).

Anatomical methods define nodes by averaging the time-series from all voxels within a given anatomical area. However, anatomical and functional spaces need not be isomorphic.

Nodes may also be represented by equally sized brain voxels. The time-series recorded from each voxel is then used to create the functional network. Alternatively, node location may be identified with the peaks or centers of mass from activation maps.

Due to the high data dimensionality (~10^5^) induced by these two methods, a pruning of the set of nodes may be required to reduce the computational cost and the amount of noise introduced by irrelevant nodes, and to ultimately improve the overall significance of the analysis. The field of data mining provides a range of feature selection techniques to perform this pruning (Liu and Motoda, 2007). This is generally accomplished by selecting or generating the features with minimum information redundancy, e.g., via principal or independent component analysis, or mutual information. Yet, it is not clear to what extent deleting or merging sets of nodes with similar dynamics eliminates physiologically relevant information.

On the one hand, the anatomo-functional space is often segmented with partitional clustering methods (Jain et al., 1999), which represent inadequate accounts of systems where the same region can simultaneously participate in different functional units. Furthermore, while a relatively small number of localized regions of interest can accurately capture distributed brain dynamics if their behavior is representative of the underlying dominant modes, reflecting the fact that brain functions range from highly localized to highly extended (Robinson, 2013), this is not guaranteed a priori and hard to check from experimental data.

Global topological properties such as small-worldness may be robust to the parcellation technique and overall number of nodes, but the quantitative aspect of these properties may be grossly affected (Zalesky et al., 2010).

Furthermore, functional activation methods require that nodes be defined in a time-varying fashion that reflects the dynamics of functional brain activity. This may lead to a fluctuating number of nodes, and care should be taken when comparing the associated topologies.

On the other hand, the brain has complex fractal structure, showing self-similarity or self-dissimilarity (Itzkovitz et al., 2005) depending on the homogeneity of pattern formation rules, and it is not clear whether information is stored locally, e.g., in single nodes or motifs, or non-locally, across widely separated units. Thus, a complete picture of brain functional activity requires that nodes be defined at different coarse-graining levels, revealing organizational principles at different levels.

## What's a link?

### Choosing the appropriate metric

Functional links are generally defined using statistical relationships between activity recorded at different brain sites or sensors, and are given either a binary or a continuous value. However, how different connectivity metrics affect the topological properties of the resulting networks and how to elect the most appropriate metric of brain activity out of the great number of available ones are still poorly understood issues.

Observed activity can be regarded as a sequence of waxing and waning synchronization and desynchronization episodes between distant brain regions. Synchronization would facilitate integrative functions, by transiently binding together spatially distributed neural populations, while desynchronization may allow the brain to flexibly switch from one coherent state to another (Varela et al., 2001).

Synchronization-based networks are configurations evaluated locally in time, with a single characteristic scale. However, functional networks may be temporally non-local. When this is the case, topological properties should not be evaluated at single snapshots, but across time. Furthermore, this approach fails to account for multiscaleness of functional brain activity, while considering synchronization at different time scales would help unveiling hierarchical neural communities (Arenas et al., 2006). Finally, hub nodes may be undetectable by single-scale synchronization metrics. Functional hubs are densely connected key components of information transmission through the network, and work as functional relay units. Multiple perturbations impair relay systems' ability to synchronize: relay units are either not synchronized or synchronized with a time delay or a complex coupling function, with the systems they mediate (Vicente et al., 2008; Gutiérrez et al., 2013). This may lead to underestimating the hubs' functional connectivity.

### Pruning links

The transformation of an all-to-all connected clique into a functional network generally requires a thresholding process, leading to an adjacency matrix, and therefore crucially depends on the threshold value.

A threshold value can either be fixed a priori (Meunier et al., 2009), or chosen after examining a range of values (Horstmann et al., 2010; van Wijk et al., 2010), or through an adaptive process (Bassett et al., 2006), e.g., by choosing its maximal value keeping the network connected (Schindler et al., 2008). A qualitatively different strategy consists in selecting the threshold level that optimizes some criterion, e.g., a data classification rate (Zanin et al., 2012).

Thresholds imply sufficiently dichotomous relations (Butts, 2009), a condition that may not always be fulfilled, particularly when the time-window within which synchronization is discretized is of the order of the average interval between synchronization events.

Pruning connections by setting a threshold has several serious, somehow interrelated, potential consequences.

First, network properties are probability distribution functions. Too high a threshold can prevent the convergence of the sample distribution to the true asymptotic one and therefore the emergence of the corresponding macroscopic property.

Second, the percentage of considered links, often set at ~5% of the total ones, may be very far from the percentage of links which optimizes data classification based on network properties, and lie in a range where classification quality is extremely vulnerable to fluctuations in network parameters (Zanin et al., 2012).

Third, a threshold biases the analysis towards certain scales and corresponding topological properties, annihilating the effect of other ones. Each network metric is strongly associated with a preferred link density. For instance, triangular motifs cannot appear in very sparse networks, while unconnected triangles disappear in very dense networks. Similarly, hub-based structures fade out for very high link densities.

Finally, however chosen, a threshold implicitly entails that there exists an optimal description level for a given system. However, the topological properties of functional brain networks qualitatively change when considering different threshold values. For instance, when considering high threshold values, brain activity appears hierarchically organized into modules with *large-world* self-similar properties, while the addition of only a few weak links is enough to render the network non-fractal and *small-world* (Gallos et al., 2012).

## Representing functional spaces with networks

Perhaps the most important and nonetheless overlooked issue in functional brain activity studies is that of defining the space within which functional activity would best be represented.

The ultimate goal of the analysis of brain function is to describe the space of functional processes (sensory, motor, cognitive, etc.) associated with neural activity, the nature of each of them, and of their interactions.

Functional brain activity is typically represented in a space isomorphic, in some sense, to the anatomical one, with nodes reflecting anatomically-related units, and links a connectivity metrics. However, connectivity may not necessarily be the best descriptor of functional activity. Rather than from connectivity, functional brain activity may for instance emerge from a collective property independent of it (Fraiman et al., 2009). Furthermore, there is no clear relationship between connectivity and transfer or processing of information.

The functional space itself could be of a more abstract nature, where networks need not be isomorphic to the topology of the brain. For example, brain activity can be represented as the motion of a diffusing macroscopic particle in a complex high-dimensional configuration space (Hsu and Hsu, 2009; Papo, 2013). Network theory may then be used to describe the phase space in which brain activity lives. Brain dynamics has been shown to be weakly non-ergodic (Bianco et al., 2007), a condition where the whole phase space is still accessible, but the time to visit certain regions may be much longer than typical experimental ones (Bouchaud, 1992). The fact that not all possible states are homogeneously populated makes the phase space look like a network, with microscopic dynamics restricted to nodes and links (Thurner, 2005).

More generally, the space of functional brain activity may take arbitrarily complex forms, comprising information with heterogeneous dimensionalities and possibly incommensurable natures. Viewing this information set as a system may then represent the most impervious step.

Identifying a system is a task in many ways akin to identifying an object. Gestalt theory (Köhler, 1929) showed that humans recognize objects as whole forms, using general grouping laws to define boundaries, constituent parts and their relationships. Complex functional spaces may not be naturally amenable to object-like grouping laws, hampering their treatment as systems and, as a consequence, the use of graph theory to study their properties. Graph theory can be generalized to a class of non-Gestaltic systems, for instance by building networks whose nodes represent features, and links quantify deviations between two features and their typical relationship within a population (Zanin et al., 2013). The structure of this generalized functional space is ultimately embedded in the topology of the reconstructed network.
